# 634 Patient Experience of Using Artificial Intelligence-Enhanced Multispectral Imaging: A Regional Burns Centre Review

**DOI:** 10.1093/jbcr/iraf019.263

**Published:** 2025-04-01

**Authors:** Miriam Nyeko-Lacek, Karl Walsh, Zeeshan Sheikh

**Affiliations:** Manchester University NHS Foundation Trust; Manchester University NHS Foundation Trust; Manchester University NHS Foundation Trust

## Abstract

**Introduction:**

Artificial intelligence (AI) has a wide range of potential uses in medicine, one of which is diagnostic aids and prediction models. Whilst the opinions of healthcare professionals on this have been considered, establishing whether this technology is acceptable to patients is also extremely important. Within our burns service we utilized a multispectral imaging device which uses AI to assess burn depth and predicts whether a burn wound will heal within 21 days. The device takes a picture of a patient’s wound and produces an computerized image as well as an image of its predictive assessment superimposed over the burn area. This can be viewed by both the clinician and the patient. We aimed to identify the patient’s experience of undergoing predictive wound assessment as well as their thoughts of using AI technology as part of a clinical consultation.

**Methods:**

Adult patients with mental full mental capacity, presenting to a UK regional burns center between 08/30/24-09/20/24 who sustained a burn wound underwent both a clinical assessment and assessment using the AI device. All patients were asked to rate their experience of the device using a 4-item questionnaire. Each question asked them to rate an aspect of using the machine on a 10-point Likert scale. Patients were asked about “speed of the process”, “how comfortable it was”, “how satisfied they would be to have further photos images taken of their wounds” and “whether using the machine helped them understand the depth of their burn” following reviewing their images on the monitor.

**Results:**

51 patients completed the questionnaire after a doctor assessed their burn wound as well as taking a picture using the AI machine. The median score for speed was 10 with an interquartile range (IQR) of 1. In terms of comfort, 84% of patients rated the device as 10, resulting in a median of 10 and IQR of 0 (range 8-10). 82% of patients gave the device a 10 for how satisfied they would be to have further images taken (range 7-10). With regards to how much the device helped them understand their burn depth, 82% of patients rated it 8 or above. This gave a median score of 10, with an IQR of 2.

**Conclusions:**

Patients found the use of multispectral imaging with AI to be a fast, comfortable and helpful tool to understand the depth of their burn wound. Most were happy for the device to be used on them in the future. Our questionnaire also demonstrates that patients are accepting of this form of technology as part of their clinical consultation.

**Applicability of Research to Practice:**

Due to the comfort and speed of the device in assessing wounds, it would potentially be even more beneficial to use the device on a paediatric cohort or in patients who may not tolerate prolonged clinical examination. Patients also noted that it aided them in making decisions regarding planning hospital visits and the potentiality of surgical intervention. Due to high satisfaction for further clinical imaging, patients could be re-evaluated in order to amend their clinical treatment plan.

**Funding for the Study:**

N/A

